# Supplementary material for: Interlayer exciton mediated second harmonic generation in bilayer MoS2
Source: Nat Commun. 2021 Nov 25;12:6894. doi: 10.1038/s41467-021-27213-8 (PMC8617052; doi:10.1038/s41467-021-27213-8)
Supplement: Supplementary file 1 — Supplementary information [file 41467_2021_27213_MOESM1_ESM.pdf]

Interlayer exciton mediated second harmonic generation in bilayer MoS_2 : *Supplementary information*

Shivangi Shree[1,2], Delphine Lagarde[1], Laurent Lombez[1], Cedric Robert[1], Andrea Balocchi[1], Kenji Watanabe[3], Takashi Taniguchi[4], Xavier Marie[1], Iann C. Gerber[1], Mikhail M. Glazov[5],[*] Leonid E. Golub[5], Bernhard Urbaszek[1],[†] and Ioannis Paradisanos[1‡]

[1] *Université de Toulouse, INSA-CNRS-UPS, LPCNO, 135 Avenue Rangueil, 31077 Toulouse, France*
[2] *Department of Physics, University of Washington, Seattle WA 9819U*
[3] *Research Center for Functional Materials, National Institute for Materials Science, 1-1 Namiki, Tsukuba 305-0044, Japan*
[4] *International Center for Materials Nanoarchitectonics, National Institute for Materials Science, 1-1 Namiki, Tsukuba 305-0044, Japan and*
[5] *Ioffe Institute, 194021 St. Petersburg, Russia*

CONTENTS

I. Sample fabrication 1

II. Experimental setup 1

III. Assignment of resonances in monolayers and trilayers 2

IV. Room temperature SHG spectroscopy of 1L, 2L, 3L MoS_2 2

V. Estimation of the SHG power, conversion efficiency and sheet-susceptibility 3

VI. Background SHG 3

VII. Polarization-resolved SHG 3

VIII. hBN nonlinear contribution 5

IX. Validation of the nonlinear processes 5

X. Theory 7
A. SHG in an ideal centrosymmetric bilayer 7
B. Electric field induced mechanism 8
1. Minimal model 8
2. Extensions of the model 10

References 11

I. SAMPLE FABRICATION

The 90 nm thick SiO_2/Si substrates were cleaned for 10 minutes in aceton and isopropanol using an ultrasonication bath and were subsequently exposed in oxygen plasma for 60 seconds. Bulk 2H MoS_2 (2D Semiconductors) was first exfoliated on Nitto Denko tape and the exfoliated areas were attached on a polydimethylsiloxane (PDMS) stamp, supported by a microscope glass slide. Monolayers, 2H-bilayers, and trilayers were identified based on the optical contrast under an optical microscope prior to transfer on the SiO_2/Si substrate. For the hBN encapsulated samples, hBN flakes were first exfoliated on a Nitto Denko tape from high quality bulk crystals [1] while the same PDMS-assisted transfer process on SiO_2/Si substrates was followed. A staircase sample of monolayers, 2H-bilayers and trilayers was susbequently transferred and capped in hBN. Between every transfer step, annealing at 150°C was applied for 60 min. Optical images of hBN encapsulated but also bare MoS_2 in SiO_2 are shown in Supplementary Fig. 1a. The thickness of the bottom hBN layers was selected to optimize the visibility of the interlayer exciton in the reflectivity spectra [2]. For the electric field device, the same process was followed including the additional transfer of few-layered graphite (FLG) flakes. The stack, starting from bottom to top consists of Si, 90 nm SiO_2, 130 nm hBN, FLG, few-layered (≈15 nm) hBN, 2L MoS_2, few-layered hBN (≈20 nm) and FLG again. The bottom and top FLG are in contact with gold (Au) electrodes to apply a potential difference and generate an electric field perpendicular to the structure. The precise sequence of the complete stack from bottom to top includes hBN/FLG/hBN/2H-MoS_2/hBN/FLG. A schematic representation of the device is shown in Supplementary Fig. 1b.

II. EXPERIMENTAL SETUP

Optical spectroscopy is performed in a home-built micro-spectroscopy set-up assembled around a closed-cycle, low vibration helium cryostat with a temperature controller (T = 4 K to 300 K). For SHG measurements we use ps pulses, generated by a tunable optical parametric oscillator (OPO) synchronously pumped by a mode-locked Ti:sapphire laser as in [3]. SHG signal is collected in reflection geometry. A combination of linear polarizers and halfwave plates allows the control of excitation and detection polarization for the polarization-resolved measurements with the setup sketched in Supplementary Fig. 2. The light is focused onto the sample at T = 4.2 K

* glazov@coherent.ioffe.ru
† urbaszek@insa-toulouse.fr
‡ paradeis@insa-toulouse.fr

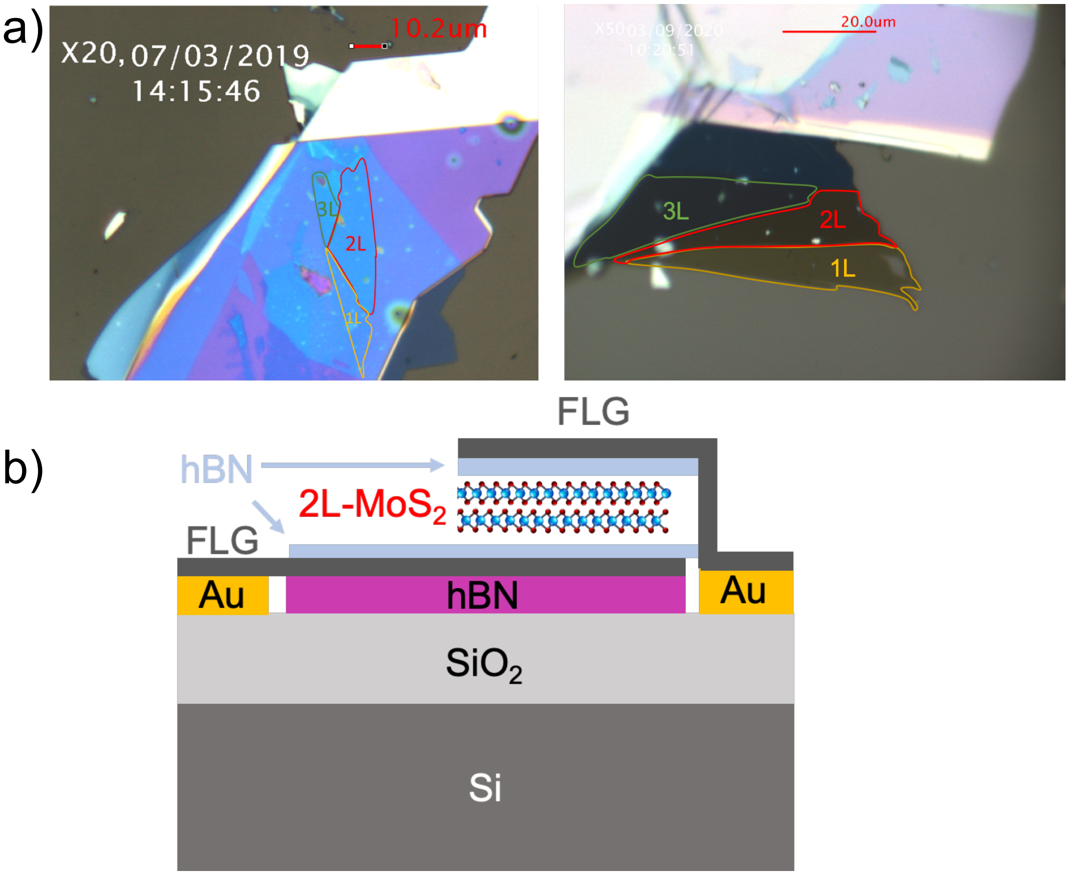

Supplementary Fig. 1. **a) Optical images of hBN encapsulated and bare MoS_2 on SiO_2. b) Schematic representation of the device to apply electric fields on MoS_2 bilayer.**

using a microscope objective (NA = 0.8). The position of the sample with respect to the focus can be adjusted with cryogenic nanopositionners. The reflected light from the sample is sent to a spectrometer with a 150 grooves per millimeter grating. The spectra are recorded by a liquid-nitrogen cooled charged coupled device (CCD) array. For low temperature white light reflectance measurements a white light source; a halogen lamp is used with a stabilized power, focused initially on a pin-hole that is imaged on the sample. The emitted and/or reflected light was dispersed in a spectrometer and detected by a Si-CCD camera. The excitation/detection spot diameter is 1 μm, i.e., smaller than the typical size of the homobilayers. We obtained differential reflectivity from reflectivity spectra as $(R_{\mathrm{ML}} - R_{\mathrm{sub}})/R_{\mathrm{sub}}$, where R_{ML} is the intensity reflection coefficient of the sample with the MoS_2 layer and R_{sub} is the reflection coefficient of the hBN/SiO_2 stack.

III. ASSIGNMENT OF RESONANCES IN MONOLAYERS AND TRILAYERS

Monolayers and trilayers have also been investigated via SHG spectroscopy. For the assignment of the different excitonic resonances in the SHG spectra, differential reflectance has also been collected from the same sample areas (compare top and bottom spectra in Supplementary Fig. 3). Besides 1s states, excited states of the A exciton can be observed in SHG and verified by reflectivity. Note that interlayer exciton states are clear in the SHG spectra of trilayers, see also [4].

IV. ROOM TEMPERATURE SHG SPECTROSCOPY OF 1L, 2L, 3L MOS$_2$

Thermal broadening effects are expected to act on the excitonic transitions at elevated temperatures, limiting the distinguishability of particular transitions, such as the interlayer excitons (IE) in bilayers. Wavelength dependent SHG experiments in 1L, 2L and 3L MoS_2 at $T = 295$ K, are presented in Supplementary Fig. 4. Our main result is that the excitonic enhancement of the SHG signal also applies to room temperature experiments, when twice the laser energy is tuned into resonance with interlayer and intralayer excitons in 1, 2, and 3L MoS_2, as can be clearly seen in Supplementary Fig. 4. We find that the excitonic resonances appear broader at

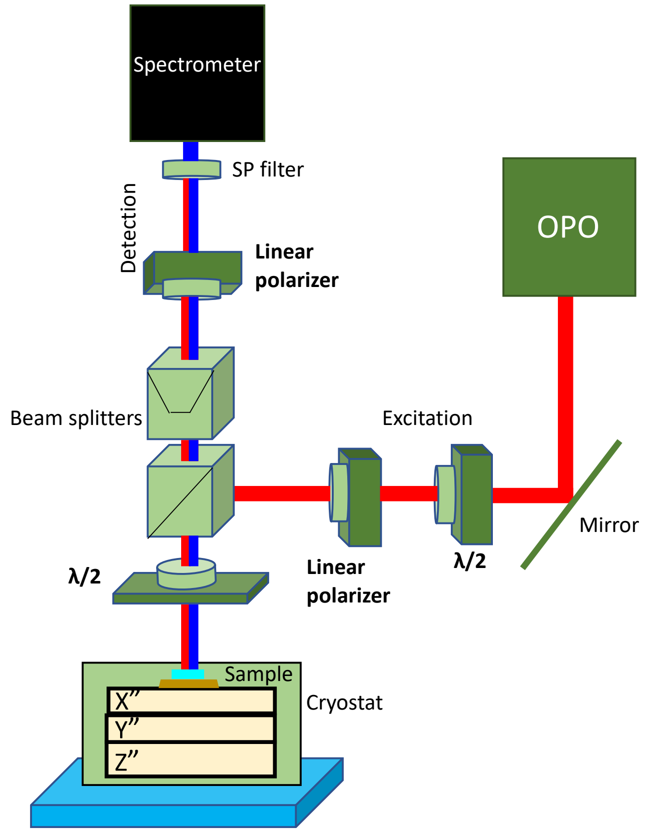

Supplementary Fig. 2. **Experimental setup used for the collection of the SHG spectra.**

$T = 295$ K than at $T = 4$ K. For instance, in 1L-MoS_2 the FWHM of the A-exciton is 35 meV at $T = 295$ K, much broader compared to 6 meV measured at $T = 4$ K. In addition, the IE in 2L-MoS_2 (middle SHG spectrum in the Supplementary Fig. 4), is now merged into the broader A2s and X states. As the resonances red-shift as a function of temperature, we notice that their relative strength is also affected possibly due to cavity effects.

V. ESTIMATION OF THE SHG POWER, CONVERSION EFFICIENCY AND SHEET-SUSCEPTIBILITY

In order to quantify the SHG power generated by the sample, optical losses due to the different components of the setup must be considered. The transmission/reflection coefficients of the optical components were taken from the manufacturers' spreadsheets for the wavelengths that lie within our SHG spectra. The optical components involved in the detection path include the objective lens (Attocube, NA = 0.81), a set of 2 glass windows that act as beam splitters, 3x silver protected mirrors (PF-20-03-P01, Thorlabs), an achromatic doublet (AC254-050-B-ML, Thorlabs), a shortpass 800 nm filter, two silver-coated mirrors in the spectrometer (Princeton Instruments, Acton SpectraPRO, SP2500), and the grating efficiency (150 g/mm, blazed at 500 nm). Combining all these characteristics we conclude that before the signal reaches the detector, we estimate optical losses for the SHG emission to be 65% (depending also on the wavelength). We also considered the quantum efficiency and gain response of our detector (LN/100 BR Excelon, Princeton Instruments). For the estimation of the sheet-SHG tensor element, $\chi^{(2)}_{sh}$, we used the following equation [5]:

$$\chi^{(2)}_{sh} = \sqrt{\frac{c^3 \varepsilon_0 f \pi r^2 t (1 + n_{eff})^6 P_{2\omega}}{S 16 \sqrt{2} \omega^2 (P_\omega)^2}}.$$

Here, c is the speed of light, ε_0 is the vacuum permittivity, $f = 80$ MHz is the laser repetition rate, $r = 1$ μm is the focused beam radius, $t = 1$ ps is the pulse duration measured with an autocorrelator at the full width at half maximum, $n_{eff} = 1.45$ is the effective refractive index of the substrate, $P_{2\omega}$ is the SHG average power (measured after estimating all the optical losses), $S = 0.94$ is a shape factor for Gaussian pulses, and P_ω is the pump average power (measured before the objective lens). In Supplementary Fig. 5 we present the estimation of the energy dependent SHG power as well as the conversion efficiency, $P_{2\omega}/P_\omega$, and $\chi^{(2)}_{sh}$ for 1L (top, red color) and 3L (bottom, black color) MoS_2 measured at $T = 295$ K. Note that there is no intrinsic $\chi^{(2)}_{sh}$ for 2L-MoS_2 because of the presence of the inversion center, see discussion in the main text and in Sec. X below. The SHG power and conversion efficiency of 2L-MoS_2 are shown in Supplementary Fig. 6. Also, we need to emphasize that the conversion efficiency is linearly dependent to the incident power, so its value is linked to the provided pump power (here 3mW power for 1L and 3L and 5mW for 2L).

VI. BACKGROUND SHG

For the gated bilayer device it is important to identify the background contribution in the SHG. Few-layered graphene (FLG), as well as hBN can provide a contribution in the SHG spectra. In Supplementary Fig. 7 we show the noise level of the CCD camera as well as the background SHG originating from hBN and FLG under zero and finite electric fields. In this measurement, we collected the SHG coming from the same stack of the gated sample, but without MoS_2. The background SHG is weak (<2 counts/second) compared to the signal from MoS_2 and it is wavelength independent. This unambiguously confirms the spectral signature of the excitonic resonances in SHG of MoS_2 bilayers.

VII. POLARIZATION-RESOLVED SHG

Polarization-resolved SHG has been applied to verify the symmetry and crystallographic orientation of MoS_2

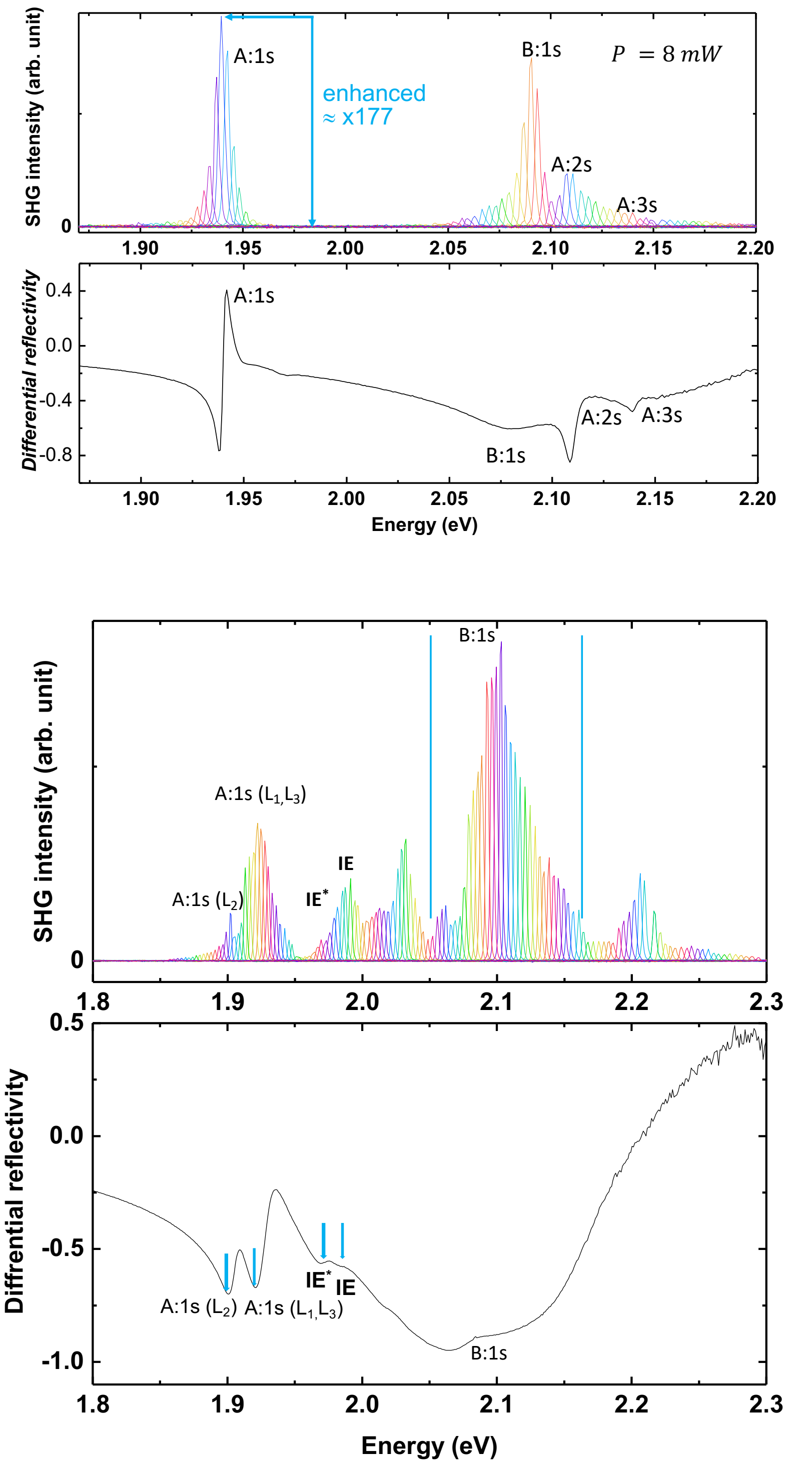

Supplementary Fig. 3. **Comparison between SHG and differential reflectivity spectra of monolayer and trilayer MoS_2.**

bilayers [6, 7]. The intensity of the SHG as a function of the polarization angle of the fundamental laser is shown in Supplementary Fig. 8. Notice that similar angle dependence is acquired when the excitation is in resonance with the A:1s and IE states. This SHG intensity is dependent on the crystallographic orientation of the sample. The symmetric 6-fold co- (XX) and cross-linear (XY) polarization over 2π reveals that any contribution from strain effects, in-plane electric fields, and low symmetry defects is negligible [8, 9].

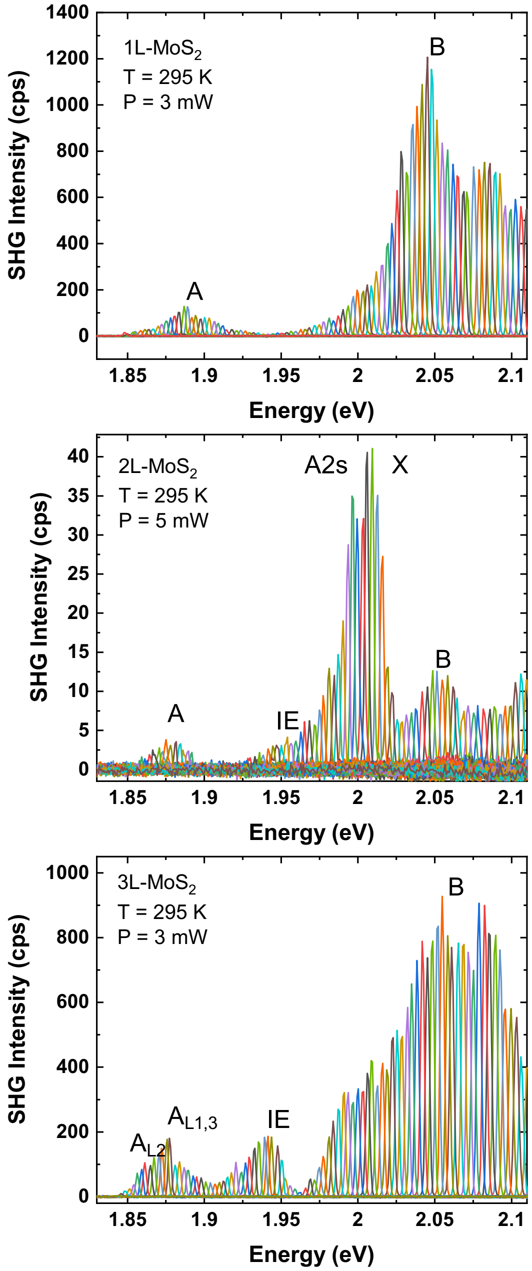

Supplementary Fig. 4. **From top to bottom: room temperature SHG spectroscopy of 1L, 2L and 3L MoS_2.**

VIII. hBN NONLINEAR CONTRIBUTION

In the encapsulated bilayers with hBN, we measure the SHG strength of the surrounding hBN in the total signal. In Supplementary Fig. 9 we compare the SHG intensity coming from the surrounding hBN with the MoS_2 bilayer when the excitation is kept constant and tuned into resonance with the A:1s state. The SHG coming from MoS_2 is two orders of magnitude stronger.

IX. VALIDATION OF THE NONLINEAR PROCESSES

Power dependent measurements have been performed in MoS_2 bilayers. In Supplementary Fig. 10 the SHG intensity is plotted as a function of laser power. The excitation energy is tuned in resonance with the A:1s state. The quadratic power law confirms the two-photon process.

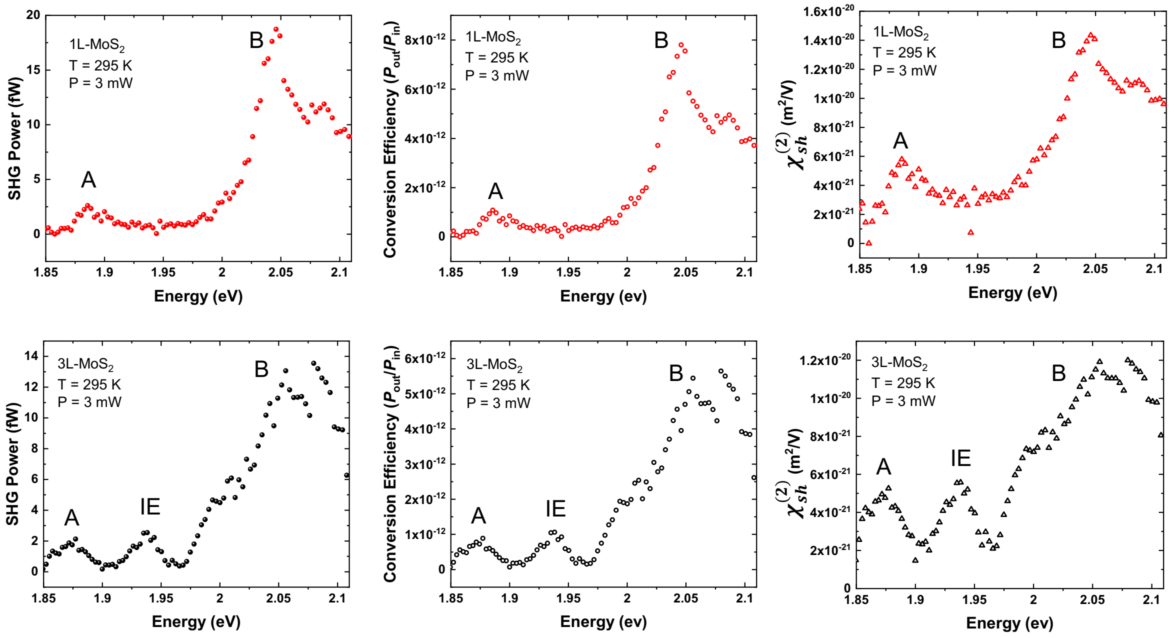

Supplementary Fig. 5. **SHG power, conversion efficiency and sheet-second order susceptibility for 1L-MoS_2 (top row – red color) and 3L-MoS_2 (bottom row – black color). The A, B and interlayer (IE) exciton resonances are labelled.**

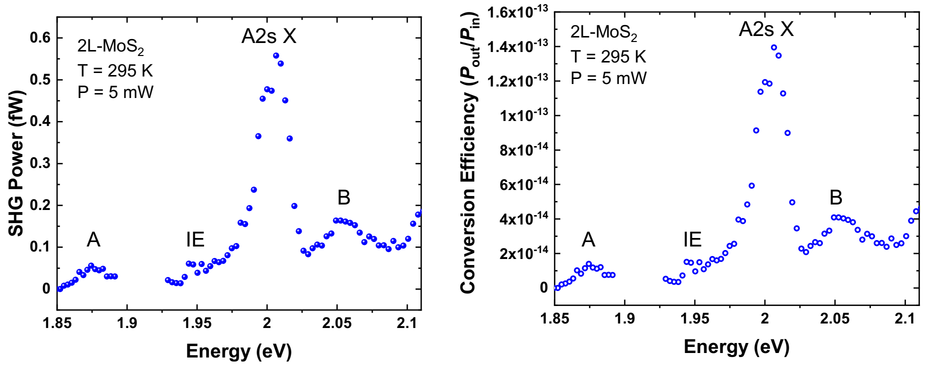

Supplementary Fig. 6. **SHG power and conversion efficiency for 2L-MoS_2. The A, B, interlayer (IE), A2s and X exciton resonances are labelled.**

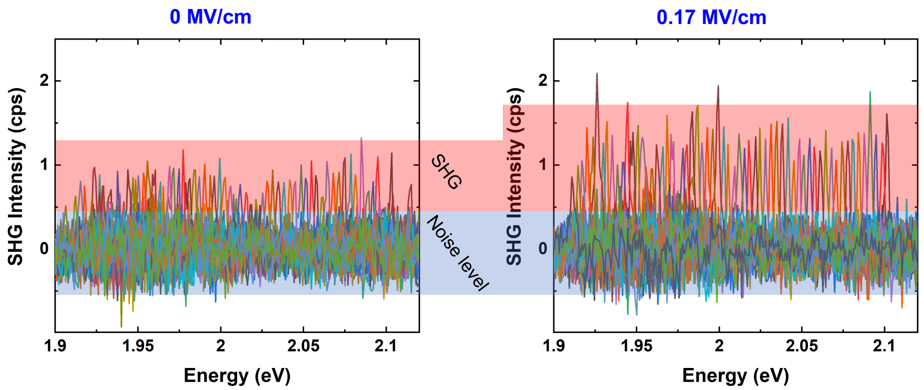

Supplementary Fig. 7. **Background SHG contribution from the device excluding MoS_2 at zero and finite electric field.**

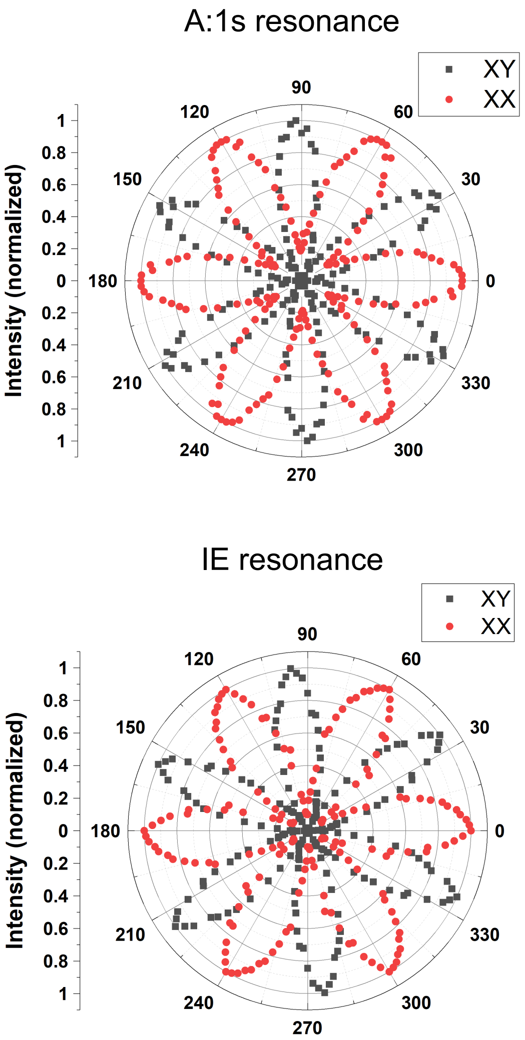

Supplementary Fig. 8. **Polarization-resolved SHG on bilayaer MoS_2 at A:1s and IE exciton resonances.**

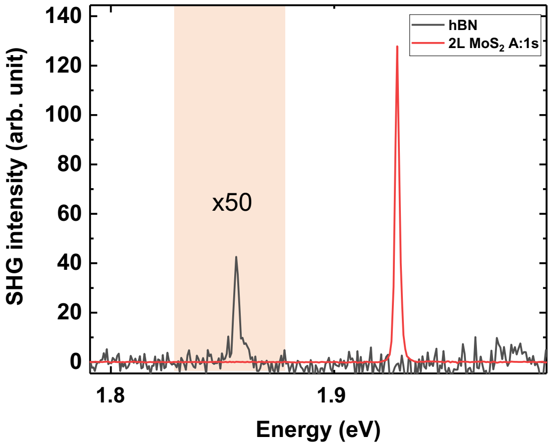

Supplementary Fig. 9. **Comparison between SHG of bilayer MoS_2 with hBN. Notice that the SHG from hBN is multiplied by a factor of 50.**

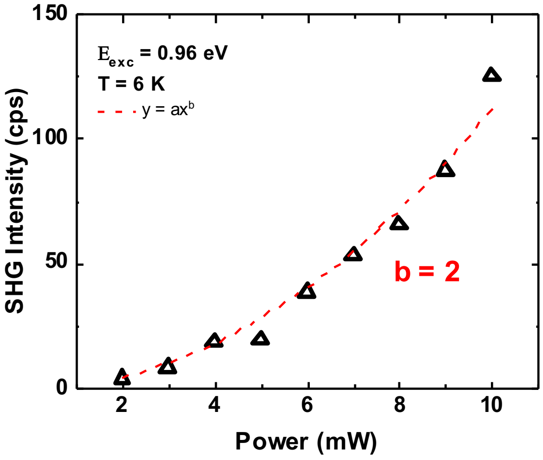

Supplementary Fig. 10. **Power dependent SHG in resonance with the A-exciton clearly demonstrates a two-photon process.**

X. THEORY

This section contains the phenomenological and microscopic theory describing the SHG in bilayer structures.

A. SHG in an ideal centrosymmetric bilayer

Bilayer has a D_{3d} point symmetry with an inversion center. For the light propagating along the normal z there is a contribution caused by the radiation wavevector q_z (quadrupolar contribution) which results from the inhomogeneity of the incident and emitted electric field distribution:

$$P_{2\omega,x} = q_z \chi' (E^2_{\omega,x} - E^2_{\omega,y}), \quad P_{2\omega,y} = -2q_z \chi' E_{\omega,x} E_{\omega,y}. \tag{1}$$

To illustrate the effect let us study the electrodynamical mechanism leading to Supplementary Eq. (1). For this mechanism we consider both monolayers forming a bilayer as electronically decoupled, the distance between the layers is a. Disregarding multiple reflections of the second harmonics radiation from both monolayers we obtain the reflected and transmitted amplitudes of the second harmonics in the form

$$E^r_{2\omega} = S\chi E^2_\omega (1 - \mathrm{e}^{i(q_{2\omega}+2q_\omega)a}), \tag{2a}$$

$$E^t_{2\omega} = S\chi E^2_\omega (\mathrm{e}^{iq_{2\omega}a} - \mathrm{e}^{i2q_\omega a}). \tag{2b}$$

Here S is the coefficient describing the propagation of the SHG field from the sample, χ is the second-order susceptibility of a monolayer [10], and $q_\Omega = \Omega n_\Omega / c$ with n_Ω being the refraction index at the frequency Ω. It is noteworthy that for a bilayer the products $q_\omega a, q_{2\omega} a \ll 1$. As a result, the intensity of the SHG from the bilayer is suppressed as compared with the monolayer by a factor of $\sim q_{2\omega} a \ll 1$, resulting in the small SHG signal from the

bilayer (see the main text for discussion). Accounting for the electronic coupling between the layers may result in additional contributions to the SHG which has the same smallness $\propto q_\omega a$.

In Supplementary Eqs. (2) we neglected the differences between the transmission coefficients of the monolayers and unity at both fundamental and double frequencies and disregarded the effect of the substrate on the fields. While the transmission coefficient of the monolayer t_ω at a fundamental frequency is very close to 1 because the incident radiation is non-resonant, the effect of the monolayers on the transmission and reflection of the second harmonic can be sizeable due to the exciton resonances. With account for multiple reflections of the second harmonics from the monolayers, we have an enhancement of both the reflected and transmitted waves. In the reflection geometry the enhancement factor [cf. Ref. [11]]

$$F_r = 1 + t_{2\omega} \frac{r_{2\omega} e^{i2q_{2\omega}a}}{1 - r_{2\omega}^2 e^{i2q_{2\omega}a}} \approx \frac{1}{1 - r_{2\omega}}, \tag{3}$$

where $r_{2\omega}$ is the reflection coefficient from the monolayer at the frequency 2ω, $t_{2\omega} = 1 + r_{2\omega}$, and in the last equation we neglected the phase acquired by the field propagating between the layers. Making use of the explicit form of the monolayer reflection coefficient we recast F_r as

$$F_r = \frac{\omega_0 - 2\omega - i(\Gamma_0 + \Gamma)}{\omega_0 - 2\omega - i(2\Gamma_0 + \Gamma)},$$

where ω_0 is the exciton resonance frequency, Γ_0 and Γ are its radiative and non-radiative damping.

The expression for the enhancement factor in the transmission geometry has somewhat more complex form

$$F_t = \frac{\mathrm{e}^{iq_{2\omega}a} \frac{t_{2\omega}}{1 - r_{2\omega}^2 \mathrm{e}^{2iq_{2\omega}a}} - \mathrm{e}^{i2q_\omega a}\left(1 + \frac{t_{2\omega} r_{2\omega} \mathrm{e}^{2iq_{2\omega}a}}{1 - r_{2\omega}^2 \mathrm{e}^{2iq_{2\omega}a}}\right)}{t_{2\omega}\mathrm{e}^{iq_{2\omega}a} - \mathrm{e}^{i2q_\omega a}}. \tag{4}$$

It follows from Supplementary Eqs. (3) and (4) that in the vicinity of the exciton resonance the enhancement due to multiple reflections is unimportant.

It is instructive to discuss the relation between the effects described above and the surface SHG effect both at oblique and normal incidence studied in a number of works, including Refs. [12–16]. It has been previously established that either the surface of a media has a reduced symmetry, e.g., [16] and allows for the SHG by itself, or the SHG arises due to the quadrupolar or magneto-dipolar transitions (qE^2 or EB mechanisms), e.g., [12]. The mechanism discussed above belongs to the second type of the effects.

To conclude this part, in ideal centrosymmetric bilayer the SHG is possible due to the effects related to the finite wavevector of the radiation and a finite width of the bilayer. Because of this, the effect is suppressed in pristine bilayer as compared to the non-centrosymmetric monolayer. We note, however, that the SHG in our bilayer samples at $F_z = 0$ is unlikely to be related to the radiation wavevector due to relatively high magnitude of the effect (although, expectedly, smaller that in the monolayers). In the following section we address the effects of the external electric field which breaks the symmetry in the bilayer, and also of other symmetry breaking perturbations which introduce an asymmetry between the individual layers forming a bilayer and can be included as a difference of bare A- and B-exciton energies in the constituting monolayers.

B. Electric field induced mechanism

1. Minimal model

Let us first consider the minimal model demonstrating the electric field induced SHG in bilayers. To that end motivated by microscopic analysis presented in Refs. [4, 17] we consider the model of two interlayer exciton states $\mathrm{IE}_{1,2}$ and two intralayer exciton states $\mathrm{B}_{1,2}$. These four states are close in energy and are effectively mixed by the hole tunneling. Let T be the (hole) tunneling constant which mixes corresponding IE and B exciton states. The Hamiltonian of the system in the presence of the electric field F_z reads (the basic states are IE_1, B_1, IE_2, B_2):

$$\mathcal{H} = \begin{pmatrix} E_{\mathrm{IE}} - \mu F_z & T & 0 & 0 \\ T^* & E_{\mathrm{B}} & 0 & 0 \\ 0 & 0 & E_{\mathrm{IE}} + \mu F_z & T \\ 0 & 0 & T^* & E_{\mathrm{B}} \end{pmatrix}. \tag{5}$$

Here μ is the indirect exciton static dipole matrix element along the structure normal (see main text for details).

For each pair of levels IE_1 & B_1 and IE_2 & B_2 we have a 2×2 Hamiltonian which is readily diagonalized as

$$E_{1,\pm}(F_z) = \frac{E_{\mathrm{IE}} - \mu F_z + E_{\mathrm{B}}}{2} \pm \sqrt{\left(\frac{E_{\mathrm{IE}} - \mu F_z - E_{\mathrm{B}}}{2}\right)^2 + |T|^2}, \tag{6a}$$

$$E_{2,\pm}(F_z) = E_{1,\pm}(-F_z), \tag{6b}$$

with the eigenfunctions

$$|1,\pm\rangle = \alpha_\pm(F_z)|\mathrm{IE}_1\rangle + \beta_\pm(F_z)|\mathrm{B}_1\rangle, \tag{6c}$$

$$|2,\pm\rangle = \alpha_\pm(-F_z)|\mathrm{IE}_2\rangle + \beta_\pm(-F_z)|\mathrm{B}_2\rangle. \tag{6d}$$

The coefficients $\alpha_\pm(F_z)$, $\beta_\pm(F_z)$ read

$$\alpha_\pm(F_z) = \frac{E_{1,\pm} - E_{\mathrm{B}}}{T^*}\beta_\pm(F_z), \tag{6e}$$

$$\beta_\pm(F_z) = \frac{1}{\sqrt{1 + \frac{(E_{1,\pm} - E_{\mathrm{B}})^2}{|T|^2}}}.$$

In order to derive compact analytical expressions we assume that the distance between the IE and B excitons $\Delta = E_B - E_{\rm IE}$ exceeds by far both $|T|$ and $|\mu F_z|$, and also that $|\mu F_z| \ll |T|$. Hence, the eigenenergies and eigenstates are

$$\widetilde{\rm IE_1}: \quad E_{\widetilde{\rm IE_1}} \approx E_{\rm IE} - \mu F_z - \frac{|T|^2}{\Delta}, \quad |\widetilde{\rm IE_1}\rangle = |{\rm IE_1}\rangle - \frac{T^*}{\Delta}\left(1 - \frac{\mu F_z}{\Delta}\right)|{\rm B_1}\rangle, \tag{7a}$$

$$\widetilde{\rm IE_2}: \quad E_{\widetilde{\rm IE_2}} \approx E_{\rm IE} + \mu F_z - \frac{|T|^2}{\Delta}, \quad |\widetilde{\rm IE_2}\rangle = |{\rm IE_2}\rangle - \frac{T^*}{\Delta}\left(1 + \frac{\mu F_z}{\Delta}\right)|{\rm B_2}\rangle, \tag{7b}$$

$$\widetilde{\rm B_1}: \quad E_{\widetilde{\rm B_1}} \approx E_{\rm B} + \frac{|T|^2}{\Delta}\left(1 - \frac{\mu F_z}{\Delta}\right), \tag{7c}$$

$$|\widetilde{\rm B_1}\rangle = \left[1 - \frac{|T|^2}{2\Delta^2}\left(1 - \frac{2\mu F_z}{\Delta}\right)\right]|{\rm B_1}\rangle + \frac{T}{\Delta}\left(1 - \frac{\mu F_z}{\Delta}\right)|{\rm IE_1}\rangle,$$

$$\widetilde{\rm B_2}: \quad E_{\widetilde{\rm B_2}} \approx E_{\rm B} + \frac{|T|^2}{\Delta}\left(1 + \frac{\mu F_z}{\Delta}\right), \tag{7d}$$

$$|\widetilde{\rm B_2}\rangle = \left[1 - \frac{|T|^2}{2\Delta^2}\left(1 + \frac{2\mu F_z}{\Delta}\right)\right]|{\rm B_2}\rangle + \frac{T}{\Delta}\left(1 + \frac{\mu F_z}{\Delta}\right)|{\rm IE_2}\rangle.$$

Let M_2 and M_1 be the two-photon and the one-photon excitation matrix elements of the B-exciton. The susceptibilities of $\rm B_{1,2}$ excitons in isolated monolayers $T = 0$ read (C is a constant) [10]

$$\chi_{\rm B,1,2} = \pm C \frac{M_1 M_2}{2\hbar\omega - E_{\rm B} + {\rm i}\Gamma_{\rm B}}. \tag{8}$$

For a bilayer at $F_z = 0$ the sum $\chi_{\rm B,1} + \chi_{\rm B,2} = 0$ signifying the presence of an inversion center.

The SHG generation results from the mixing of those states with B-excitons. Generally, the susceptibility can be written as

$$\chi = -CM_1M_2 \sum_{j=1}^{2} \sum_{\pm} (-1)^j \frac{|\beta_\pm(F_z)|^2}{2\hbar\omega - E_{j,\pm}(F_z) + {\rm i}\Gamma_{j,\pm}}. \tag{9}$$

As a result, for the weak tunneling and field we obtain from Supplementary Eqs. (7)

$$\widetilde{\rm IE_1}: \quad \chi_{\rm IE_1} = \frac{|T|^2}{\Delta^2}\left(1 - \frac{2\mu F_z}{\Delta}\right)\frac{CM_1M_2}{2\hbar\omega - E_{\rm IE} + \mu F_z + \frac{|T|^2}{\Delta} + {\rm i}\Gamma_{\rm IE}}, \tag{10a}$$

$$\widetilde{\rm IE_2}: \quad \chi_{\rm IE_2} = -\frac{|T|^2}{\Delta^2}\left(1 + \frac{2\mu F_z}{\Delta}\right)\frac{CM_1M_2}{2\hbar\omega - E_{\rm IE} - \mu F_z + \frac{|T|^2}{\Delta} + {\rm i}\Gamma_{\rm IE}}, \tag{10b}$$

$$\widetilde{\rm B_1}: \quad \chi_{\rm B_1} = \left[1 - \frac{|T|^2}{2\Delta^2}\left(1 - \frac{2\mu F_z}{\Delta}\right)\right]\frac{CM_1M_2}{2\hbar\omega - E_{\rm B} - \frac{|T|^2}{\Delta}\left(1 - \frac{\mu F_z}{\Delta}\right) + {\rm i}\Gamma_{\rm B}}, \tag{10c}$$

$$\widetilde{\rm B_2}: \quad \chi_{\rm B_2} = -\left[1 - \frac{|T|^2}{2\Delta^2}\left(1 + \frac{2\mu F_z}{\Delta}\right)\right]\frac{CM_1M_2}{2\hbar\omega - E_{\rm B} - \frac{|T|^2}{\Delta}\left(1 + \frac{\mu F_z}{\Delta}\right) + {\rm i}\Gamma_{\rm B}}. \tag{10d}$$

The total susceptibility

$$\chi = \chi_{\rm IE_1} + \chi_{\rm IE_2} + \chi_{\rm B_1} + \chi_{\rm B_2}. \tag{11}$$

It vanishes at $F_z = 0$. At $F_z \neq 0$ the inversion symmetry is broken and effect arises both due to the energy shifts of the excitons and due to the linear in F_z contributions to the oscillator strength.

To get better insight into the SHG in bilayer materials and for the comparison with available experiments it is instructive to analyze the situation where the electric field is so weak such that $|\mu F_z| \ll \Gamma_{\rm B,IE,...}$. The suscep-

tibility in the vicinity of the IE excitons takes the form

$$\chi_{\rm IE} = \chi_{{\rm IE}_1} + \chi_{{\rm IE}_2} = -\frac{2\mu F_z}{2\hbar\omega - E_{\rm IE} + \frac{|T|^2}{\Delta} + {\rm i}\Gamma_{\rm IE}} \frac{CM_1M_2|T|^2}{\Delta^2} \times \left(\frac{2}{\Delta} + \frac{1}{2\hbar\omega - E_{\rm IE} + \frac{|T|^2}{\Delta} + {\rm i}\Gamma_{\rm IE}}\right). \quad (12)$$

For reasonable $\Delta > \Gamma_{\rm IE}$ the second term in parentheses dominates in the vicinity of the resonance. Thus, equation (12) reduces to

$$\chi_{\rm IE} = -\frac{2\mu F_z}{\left(2\hbar\omega - E_{\rm IE} + \frac{|T|^2}{\Delta} + {\rm i}\Gamma_{\rm IE}\right)^2} \frac{CM_1M_2|T|^2}{\Delta^2}, \quad (13)$$

which is equivalent to Supplementary Eq. (2) of the main text with the notation $C_2|\Phi_{{\rm B}:1s}(0)|^2 = CM_1M_2$, cf. Ref. [10]. Similar expression can be derived describing the susceptibility in the vicinity of the B excitons:

$$\chi_{\rm B} = \chi_{{\rm B}_1} + \chi_{{\rm B}_2} = -\frac{2\mu F_z}{2\hbar\omega - E_{\rm B} - \frac{|T|^2}{\Delta} + {\rm i}\Gamma_{\rm B}} \frac{CM_1M_2|T|^2}{\Delta^2} \times \left(\frac{1}{\Delta} - \frac{1}{2\hbar\omega - E_{\rm B} - \frac{|T|^2}{\Delta} + {\rm i}\Gamma_{\rm B}}\right). \quad (14)$$

2. *Extensions of the model*

The deficiency of the model above is the absence of the effect on A-excitons and also the neglect of the mixing of intralayer excitons due to the tunneling and symmetry-breaking perturbations.

Let us first include exciton tunneling as a whole described by the matrix element $T_{\rm B}$ in the Hamiltonian, which tends to form symmetric and antisymmetric combinations of B-excitons as well as symmetry breaking between the MLs in bilayer resulting in the splitting of B-excitons with $E_{{\rm B},1} - E_{{\rm B},2} = \delta E_{\rm B}$ (note that $\delta E_{\rm B}$ contains the real and imaginary parts, the former describes the effect of the asymmetry on the positions of the exciton resonances and the latter accounts for different non-radiative dampings of excitons):

$$\mathcal{H} = \begin{pmatrix} E_{\rm IE} - \mu F_z & T & 0 & 0 \\ T^* & E_{{\rm B},1} & 0 & T_{\rm B} \\ 0 & 0 & E_{\rm IE} + \mu F_z & T \\ 0 & T_{\rm B}^* & T^* & E_{{\rm B},2} \end{pmatrix}. \quad (15)$$

We focus at the reasonable relation between the parameters of the system with the electric field being low as compared with the broadening of the indirect exciton, $|\mu F_z| \ll \Gamma_{\rm IE} \ll \Delta$, but, on the other hand, sufficiently high so that $|\mu F_z| \gg |TT_{\rm B}/\Delta|$, $\delta E_{\rm B}|T|^2/\Delta^2$. Under these approximations the $T_{\rm B}$ and $\delta E_{\rm B}$ do not affect the IE exciton and its susceptibility is given by Supplementary Eq. (12).

The effective Hamiltonian describing the doublet of B-excitons in the second-order in T and lowest order in μF_z, δE_B reads

$$\mathcal{H} = \begin{pmatrix} E_{{\rm B},1} + \mu F_z \frac{|T|^2}{\Delta^2} + \frac{|T|^2}{\Delta} & T_{\rm B} \\ T_{\rm B}^* & E_{{\rm B},2} - \mu F_z \frac{|T|^2}{\Delta^2} + \frac{|T|^2}{\Delta} \end{pmatrix}. \quad (16)$$

Furthermore, in a weak asymmetry limit where $|T_B| \gg \delta E_B, |\mu F_z||T|^2/\Delta^2$, the Hamiltonian (16) in the basis of $(|{\rm B}_1\rangle \pm |{\rm B}_2\rangle)/\sqrt{2}$ states transforms to (we assume that the diagonal terms $|T|^2/\Delta$ are included in $E_{\rm B}$)

$$\mathcal{H} = \begin{pmatrix} E_{\rm B} + T_{\rm B} & \delta E_B + \mu F_z \frac{|T|^2}{\Delta^2} \\ \delta E_B + \mu F_z \frac{|T|^2}{\Delta^2} & E_{\rm B} - T_{\rm B} \end{pmatrix}, \quad (17)$$

whose eigenstates are:

$$|{\rm B}_+\rangle: \quad E_{{\rm B}_+} = E_{\rm B} + T_{\rm B}, \quad (18)$$

$$|{\rm B}_+\rangle = \frac{|{\rm B}_1\rangle + |{\rm B}_2\rangle}{\sqrt{2}} + \frac{\delta}{2T_{\rm B}} \frac{|{\rm B}_1\rangle - |{\rm B}_2\rangle}{\sqrt{2}},$$

$$|{\rm B}_-\rangle: \quad E_{{\rm B}_-} = E_{\rm B} - T_{\rm B}, \quad (19)$$

$$|{\rm B}_+\rangle = \frac{|{\rm B}_1\rangle - |{\rm B}_2\rangle}{\sqrt{2}} - \frac{\delta}{2T_{\rm B}} \frac{|{\rm B}_1\rangle + |{\rm B}_2\rangle}{\sqrt{2}},$$

with $\delta = \delta E_{\rm B} + \mu F_z|T|^2/\Delta^2$. As a result, the non-linear susceptibility in the spectral range of B-excitons reads

$$\chi = \frac{\delta}{2T_B} CM_1M_2 \times \left(\frac{1}{2\hbar\omega - E_{\rm B} - T_{\rm B} + {\rm i}\Gamma_B} - \frac{1}{2\hbar\omega - E_{\rm B} + T_{\rm B} + {\rm i}\Gamma_B}\right) \approx \left(\delta E_{\rm B} + \frac{\mu F_z|T|^2}{\Delta^2}\right) \frac{CM_1M_2}{(2\hbar\omega - E_{\rm B} + {\rm i}\Gamma_{\rm B})^2 - T_{\rm B}^2} \quad (20)$$

Very similar treatment can be made for A-exciton assuming that it is mixed with the IE(B) exciton, which follows from the spin conservation rule. Making natural changes: $T_{\rm B}$ to the tunneling matrix element of the A-exciton $T_{\rm A}$, Δ to the splitting $\Delta' = E_{\rm A} - E_{\rm IE(B)}$, and $\delta E_{\rm B}$ to the A-exciton splitting $E_{{\rm A},1} - E_{{\rm A},2} = \delta E_{\rm A}$, we obtain

$$\chi_{\rm A} = \delta' \frac{C'M_1M_2}{(2\hbar\omega - E_{\rm A} + {\rm i}\Gamma_{\rm A})^2 - T_{\rm A}^2}. \quad (21)$$

Here $\delta' = \delta E_{\rm A} + \mu F_z|T_{\rm A}|^2/\Delta'^2$. As above, $\delta E_{\rm A}$ includes both the real and imaginary parts, the latter accounts for the asymmetry of the dampings for the monolayers forming a bilayer. It follows from Supplementary Eq. (21) that the effect of the electric field on A-exciton is weaker than on the IE. It follows from atomistic calculations that the A-excitons can be mixed with interlayer exciton

IE_1 via the spin-flip electron tunneling. This effect can be included in our model in a same way, resulting in susceptibilities similar to Supplementary Eqs. (12) and (14) derived for the mixing of the IE- and B-excitons.

---

[1] Taniguchi, T. & Watanabe, K. Synthesis of high-purity boron nitride single crystals under high pressure by using Ba–BN solvent. *Journal of crystal growth* **303**, 525–529 (2007).

[2] Robert, C. *et al.* Optical spectroscopy of excited exciton states in mos 2 monolayers in van der waals heterostructures. *Physical Review Materials* **2**, 011001 (2018).

[3] Wang, G. *et al.* Giant enhancement of the optical second-harmonic emission of WSe_2 monolayers by laser excitation at exciton resonances. *Physical review letters* **114**, 097403 (2015).

[4] Leisgang, N., Shree, S., Paradisanos, I. *et al.* Giant stark splitting of an exciton in bilayer MoS_2. *Nat. Nanotechnol.* **15**, 901–907 (2020).

[5] Woodward, R. *et al.* Characterization of the second-and third-order nonlinear optical susceptibilities of monolayer mos2 using multiphoton microscopy. *2D Materials* **4**, 011006 (2016).

[6] Li, Y. *et al.* Probing symmetry properties of few-layer MoS_2 and h-BN by optical second-harmonic generation. *Nano letters* **13**, 3329–3333 (2013).

[7] Malard, L. M., Alencar, T. V., Barboza, A. P. M., Mak, K. F. & de Paula, A. M. Observation of intense second harmonic generation from MoS_2 atomic crystals. *Phys. Rev. B* **87**, 201401 (2013).

[8] Cunha, R. *et al.* Second harmonic generation in defective hexagonal boron nitride. *Journal of Physics: Condensed Matter* **32**, 19LT01 (2020).

[9] Mennel, L. *et al.* Optical imaging of strain in two-dimensional crystals. *Nature communications* **9**, 1–6 (2018).

[10] Glazov, M. *et al.* Intrinsic exciton-state mixing and nonlinear optical properties in transition metal dichalcogenide monolayers. *Physical Review B* **95**, 035311 (2017).

[11] Fang, H. H. *et al.* Control of the exciton radiative lifetime in van der waals heterostructures. *Phys. Rev. Lett.* **123**, 067401 (2019).

[12] Bloembergen, N., Chang, R. K., Jha, S. S. & Lee, C. H. Optical second-harmonic generation in reflection from media with inversion symmetry. *Phys. Rev.* **174**, 813–822 (1968).

[13] Shen, Y. Surface properties probed by second-harmonic and sum-frequency generation. *Nature* **337**, 519–525 (1989).

[14] Guyot-Sionnest, P., Chen, W. & Shen, Y. R. General considerations on optical second-harmonic generation from surfaces and interfaces. *Phys. Rev. B* **33**, 8254–8263 (1986).

[15] Sipe, J. E., Moss, D. J. & van Driel, H. M. Phenomenological theory of optical second- and third-harmonic generation from cubic centrosymmetric crystals. *Phys. Rev. B* **35**, 1129–1141 (1987).

[16] Maki, J. J., Kauranen, M. & Persoons, A. Surface second-harmonic generation from chiral materials. *Phys. Rev. B* **51**, 1425–1434 (1995).

[17] Lorchat, E. *et al.* Excitons in bilayer MoS_2 displaying a colossal electric field splitting and tunable magnetic response. *Phys. Rev. Lett.* **126**, 037401 (2021).
